# Editorial: Exploring macrophage metabolic adaptations to bacterial infection: pathways and immune responses

**DOI:** 10.3389/fcimb.2025.1730767

**Published:** 2025-11-17

**Authors:** Marc Herb, Alexander Gluschko, Raja Ganesan, Nirmal Robinson

**Affiliations:** 1 Faculty of Medicine and University Hospital of Cologne, Institute for Medical Microbiology, Immunology and Hygiene, Cologne, Germany; 2 Institute for Molecular Immunology, CECAD Research Center, University Hospital Cologne, Cologne, Germany; 3 Cluster of Excellence in Cellular Stress Responses in Aging-Associated Diseases (CECAD), University of Cologne, Cologne, Germany; 4 Center for Cancer Biology, University of South Australia and SA Pathology, Adelaide, SA, Australia; 5 Adelaide Medical School, Faculty of Health and Medical Sciences, The University of Adelaide, Adelaide, SA, Australia

**Keywords:** immunometabolism, macrophages, bacterial infection, antioxidative responses, keyword

Macrophages undergo profound metabolic changes during bacterial infections. This metabolic reprogramming is recognized as central to their function and is the focus of this Research Topic. It brings together original research articles, reviews, and perspectives that illuminate how intracellular bacterial pathogens manipulate the host metabolism to their advantage.

Peng et al. identified Rv3737 of M. tb., a transmembrane protein and a homolog of threonine transporter, as a novel virulence factor. It promotes macrophage polarization toward an anti-inflammatory state, aiding bacterial survival. Ju et al., performed comprehensive proteomic analyses to distinguish between smear-positive and smear-negative tuberculosis, revealing distinct immune activation and lipid metabolism that could open avenues for precision diagnostics and targeted therapies.

Autophagy is a process by which cells degrade damaged organelles and protein aggregates to maintain metabolic and cellular homeostasis. Hos et al. reveal that the inflammasome complex (ASC specks) degradation through autophagy is regulated by p62, establishing it as a metabolic mediator between autophagy and inflammation. Wang et al. explored foamy macrophages in leprosy, showing that lipid-induced changes enhance immune activation, with CXCL13 playing a central role in lymphocyte recruitment. Perez-Toledo and Llibre compared metabolic responses to M.tb and S.t. infections, noting that, while both pathogens induce glycolysis and lipid synthesis, they exploit these pathways differently: M.tb utilizes lipids as nutrients, while S.t. prefers carbohydrates. Autophagy again emerges as a pivotal process, with pathogen-specific outcomes.

Ting's review on Hypoxia-Inducible Factors (HIFs) and nuclear factor erythroid 2-related factor 2 (NRF2) underscores their key role in macrophage metabolism. HIF-1α drives glycolysis under hypoxia, while NRF2 regulates redox balance and enhances phagocytosis and lysosomal fusion by upregulating the phagocytic receptor Macrophage Receptor with Collagenous Structure (MARCO). These regulators are modulated by metabolites like itaconate and p62, forming a complex network of immune control. Deramaudt et al. reinforces the therapeutic potential of targeting NRF2 by demonstrating that CDDO-Me, a NRF2 activator, lowers bacterial burden both *in vitro* and *in vivo*.

Jiang and Huiang's mini review on extracellular vesicles (EVs) reveal their dual role in bacterial communication and immune modulation. EVs from different bacteria elicit varied macrophage responses, influencing inflammation, cell death, and even antiviral defenses. Engineered EVs hold promise for targeted immunotherapies.

Together, these studies highlight the intricate metabolic choreography between macrophages and pathogens. Understanding these dynamics opens new avenues for precision medicine, offering strategies to harness or modulate immune metabolism in the fight against infectious diseases.

